# Surgical mortality in patients *in extremis*: futility in emergency abdominal surgery

**DOI:** 10.1186/s12893-022-01897-1

**Published:** 2023-01-27

**Authors:** Camilo Ramírez-Giraldo, Andrés Isaza-Restrepo, Juan Camilo García-Peralta, Juliana González-Tamayo, Milcíades Ibáñez-Pinilla

**Affiliations:** 1 Hospital Universitario Mayor, Méderi, Calle 24 #29-45, Bogotá, Colombia; 2 Universidad del Rosario, Bogotá, Colombia

**Keywords:** Futility, Mortality, Emergency laparotomy, Surgical ethics, Risk factors

## Abstract

**Background:**

The number of older patients with multiple comorbidities in the emergency service is increasingly frequent, which implies the risk of incurring in futile surgical interventions. Some interventions generate false expectations of survival or quality of life in patients and families and represent a negligible therapeutic benefit in patients whose chances of survival are minimal. In order to address this dilemma, we describe mortality in a cohort of patients undergoing emergency laparotomy with a risk ≥ 75% per the ACS NSQIP Surgical Risk Calculator.

**Methods:**

A retrospective observational study was designed to analyze postoperative mortality and factors associated with postoperative mortality in a cohort of patients undergoing emergency laparotomy between January 2018 and December 2021 in a high-complexity hospital who had a mortality risk ≥ 75% per the ACS NSQIP Surgical Risk Calculator.

**Results:**

A total of 890 emergency laparotomies were performed during the study period, and 50 patients were included for the analysis. Patient median age was 82.5 (IQR: 18.25) years old and 33 (66.00%) were male. The most frequent diagnoses were mesenteric ischemia 21 (42%) and secondary peritonitis 18 (36%). Mortality in the series was 92%. Twenty-four (54.34%) died within the first 24 h of the postoperative period; 11 (23.91%) within 72 h and 10 (21.73%) within 30 days. APACHE II and SOFA scores were statistically significantly higher in patients who died.

**Conclusions:**

All available tools should be used to make decisions, with the most reliable and objective information possible, and be particularly vigilant in patients at extreme risk (mortality risk greater than 75% according to ACS NSQIP Surgical Risk Calculator) to avoid futility and its consequences. The available information should be shared with the patient, the family, or their guardians through an assertive and empathetic communication strategy. It is necessary to insist on a culture of surgical ethics based on reflection and continuous improvement in patient care and to know how to accompany them in order to have a proper death.

**Supplementary Information:**

The online version contains supplementary material available at 10.1186/s12893-022-01897-1.

## Introduction

Older patients with multiple comorbidities attending medical consultation with emergency surgical conditions are becoming increasingly common. While the overall mortality risk for surgical procedures ranges from 1.5 to 9.8%, the mortality rate after emergency laparotomy in patients over 65 years old is estimated to range from 15 to 44%, with variability dependent on perioperative conditions [1, 2]. Some statistics also show that 31.9% of patients over 65 years old had some surgical procedure in the last year of their life, and that about 1 in 10 had a surgical procedure in the last week of their life [3]. These data invite to reflect on whether the indication for these procedures in critically ill patients prioritized quality of life or length of life.

In the context of critically ill patients, indication for emergency laparotomy may induce the surgeon to incur into “futile” interventions. In patients with non-survivable conditions or a life-limiting disease, surgical intervention may provide negligible therapeutic benefit and falsely raise hope of survival and return to a better quality of life. In addition, the surgeon’s required invested effort and the resources consumed in an emergency laparotomy may be futile. Despite this and the frequency with which medical professionals encounter this dilemma, there is little evidence available and no robust recommendations to guide decisions about whether or not to perform a surgical procedure in this context [3, 4].

Semantically, futility refers to something useless or of little importance, and in a clinical setting, there is a difference between quantitative and qualitative futility. Quantitative futility is understood as a synonym for physiological futility and refers to the low probability of success for the procedure; however, most authors do not commit to quantitative thresholds with the available scales that qualify a procedure as futile, and as a result such a definition will depend on the individual patient’s context. Qualitative futility refers to the probability that a given treatment will result in an unacceptable quality of life or functional status. This concept is even more controversial, due to the different conceptions of what is considered acceptable or unacceptable, and given the wide range of cultural appreciations in this regard, it is not easy to establish this type of futility in a standard manner [5, 6].

Death occurring in the immediate postoperative period suggests futile previous interventions unable to significantly prolong survival or improve clinical outcome. Futility then represents a futile effort that increases the suffering of the patient, their family, and the costs of hospital care [4], but the decision as to whether an intervention may be futile represents a complex problem. Several investigations have been proposed to estimate risk of mortality in surgical patients and multiple scales have been designed for this purpose. They aide and facilitate decisions, provide information to patients and families, and warn about the possibility of incurring in futile procedures [4].

To calculate surgical outcomes such as risk of complications, surgical site infection, thromboembolic events, hospital stay, or mortality depending on the procedure to be performed and patient characteristics, one of the tools available is the ACS NSQIP Surgical Risk Calculator. It is expected that results obtained from the calculator will allow surgeons and patients to make better-informed decisions, although it does not propose cut-off points to contraindicate a procedure [7]. Patients with a mortality risk ≥ 75% according to this scale are considered of extreme risk [8].

This study aims to provide information on postoperative mortality in patients identified as extreme risk according to the ACS NSQIP Surgical Risk Calculator and the relevance for its consideration when indicating an emergency surgical procedure.

## Patients and methods

### Study design

A retrospective cohort observational study was designed. The medical history records of all patients undergoing emergency laparotomy at the Hospital Universitario Mayor-Méderi, a high-complexity hospital, between January 2018 and December 2021 were reviewed. Emergency laparotomy was defined by the surgeon on duty at his/her discretion based on clinical, paraclinical, and imaging findings and institutional guidelines, and was performed with prior informed consent of the patient or the patient’s guardian.

Cases with a mortality risk ≥ 75% according to the ACS NSQIP Surgical Risk Calculator were identified and included for analysis. Cases under 18 years old, reinterventions, trauma patients and those without institutional follow-up were excluded. Follow-up was performed until death or during the first 30 postoperative days. Variables collected included patients’ demographic characteristics: body mass index; comorbidities (diabetes mellitus, arterial hypertension, chronic obstructive pulmonary disease, chronic kidney disease, heart failure, recent acute myocardial infarction [< 6 months], liver disease, oncological disease, terminal oncological disease, stroke with sequelae); preoperative vasopressor and/or mechanical ventilation requirement; preoperative laboratories; indexes and scales (Charlson Comorbidity Index, Barthel Index, APACHE II Scale, SOFA, Glasgow, Celiotomy Score, ACS NSQIP Surgical Risk Calculator, Fragility Index); intraoperative findings; mortality and time of mortality if it occurred during the first 30 days of the postoperative period. Variables were collected in an anonymous database.

### Statistical analysis

Descriptive statistics were performed for demographic, clinical, paraclinical, intraoperative findings, and mortality variables. Categorical variables were described as proportions and continuous variables as medians with their respective interquartile range (IQR). A bivariate analysis was performed, with Fisher's exact test in the case of categorical variables and the Mann–Whitney test in the case of continuous variables between patients who survived and those who did not at a 24 h and 30 days cut-off point, considering a *p* < 0.05 as a statistically significant difference. Subsequently, a regression model was performed to evaluate variables associated with mortality. The entire analysis was performed in STATA^®^17, considering a statistically significant *p* < 0.05.

This study is considered of minimal risk because it does not involve interventions on patients and was reviewed and approved by the Ethics Committee of the Universidad del Rosario (number DVO005 1998 -CV1569). We followed STROBE guidelines to report this study [9].

## Results

Between January 2018 and December 2021, 890 emergency laparotomies were performed at the Hospital Universitario Mayor-Méderi. Records of 50 patients who had a mortality risk ≥ 75% according to ACS NSQIP Surgical Risk Calculator were identified and included for the analysis. The median age was 82.5 (IQR: 18.25) years old and 33 (66%) were male. The demographic, paraclinical, and clinical characteristics of the patients and the differences between them according to whether they died or survived at 30 days are shown in Table 1. One of the patients included in the analysis did not have an arterial blood gas test, other than that, no other variables were missing.

**Table 1:** Comparison of demographic, clinical, and surgical characteristics of patients undergoing emergency laparotomy with a mortality risk greater than 75% according to the ACS -NSQIP Surgical Risk Calculator during the first at 30 days of outcome

	N (%)	Dead at 30 days n = 46 (%)	Alive at 30 days n = 4 (%)	*P*-value
Age (median) (IQR) years	82.5 (18.25)	82.5 (18.50)	84 (10.25)	0.19*
Sex				
Female	17 (34.00)	15 (32.61)	2 (50.00)	0.48
Male	33 (66.00)	31 (67.39)	2 (50.00)	
Body Mass Index (median) (IQR) kg/m^2^	25 (9.47)	25.55 (8.02)	16.75 (9.6)	0.01*
Comorbidities				
Diabetes mellitus	11 (22.00)	10 (21.74)	1 (25.00)	0.64
Arterial hypertension	41 (82.00)	37 (80.43)	4 (100.00)	0.44
Chronic obstructive pulmonary disease with O_2_ requirement	17 (34.00)	17 (36.96)	0 (0.00)	0.17
Non-O_2_ requirement obstructive pulmonary disease	4 (8.00)	2 (4.35)	2 (50.00)	**0.02**
Chronic kidney disease stage 5	9 (18.00)	8 (17.39)	1 (25.00)	0.17
Heart failure	15 (30.00)	13 (28.26)	2 (50.00)	0.34
Acute myocardial infarction < 6 months	2 (4.00)	2 (4.35)	0 (0.00)	0.84
Oncologic disease	12 (24.00)	10 (21.74)	2 (50.00)	0.24
Terminal oncologic disease	7 (14.00)	6 (13.04)	1 (25.00)	0.46
Stroke with sequelae	5 (10.00)	5 (10.87)	0 (0.00)	0.64
Charlson Comorbidity Index (median) (IQR) points	6 (2.25)	6 (2)	4.5 (4.75)	0.46
Barthel Index (median) (IQR) points	65 (31.25)	65 (30)	42.5 (42.5)	0.01*
Fragility Index (median) (IQR) points	4.5 (3.00)	4 (3.00)	5.5 (1.75)	0.04*
Vasopressor				
No	15 (30.00)	11 (23.91)	4 (100.00)	0.00*
Yes	35 (70.00)	35 (76.09)	0 (0.00)	
Mechanical ventilation				
No	23 (46.00)	19 (41.30)	4 (100.00)	0.03*
Yes	27 (54.00)	27 (58.70)	0 (0.00)	
Glasgow (median) (IQR) points	9 (7.00)	8 (7.00)	13.5 (1.75)	0.01*
APACHE II (median) (IQR) mortality	36 (15.5)	31.5 (14.5)	14.5 (9.25)	0.00*
SOFA (median) (IQR) points	10 (7.00)	11 (7.00)	6 (2.75)	0.02*
Celiotomy (median) (IQR) points	10 (7.50)	10 (8.50)	10.5 (3.25)	0.40
Laboratories (median) (IQR)				
Leukocytes (× 10^3^)	13.45 (10.65)	13.45 (10.87)	13.37 (12.41)	0.37*
Hemoglobin (mg/dl)	11.1 (4.22)	11.10 (4.15)	12.3 (7.3)	0.46*
Platelets (× 10^3^)	170 (184.5)	158 (170)	300 (443)	0.08*
Creatinine (mg/dl)	2.01 (2.23)	2.04 (2.22)	1.71 (6.44)	0.39*
Sodium (mEq/L)	140 (6.5)	140 (6.75)	137 (10.5)	0.04*
Potassium (mEq/L)	4.71 (1.34)	4.70 (1.25)	5.69 (1.75)	0.06*
Glycaemia (mg/dl)	144 (94)	138 (91.5)	206 (165.5)	0.02*
Lactate (mol/L)	5.4 (5.9)	4.5 (6.59)	5.85 (1.85)	0.23*
HCO_3_	16 (8.4)	16 (8.75)	15.5 (5.42)	0.34*
pH	7.30 (0.26)	7.30 (0.28)	7.39 (0.11)	0.09*
Pa/FiO_2_	178 (99.5)	175 (97.00)	309.50 (129.75)	0.00*
Intraoperative findings				
No finding	4 (8.00)	2 (4.35)	2 (50.00)	**0.02**
Overall ischemia	5 (10.00)	5 (10.87)	0 (0.00)	0.64
Ischemia of an isolated segment	16 (32.00)	14 (30.43)	2 (50.00)	0.38
Colitis	3 (6.00)	3 (6.52)	0 (0.00)	0.77
GI Bleeding	9 (18.00)	9 (19.57)	0 (0.00)	0.44
Peptic ulcer	4 (8.00)	4 (8.70)	0 (0.00)	0.70
Peritonitis	18 (36.00)	18 (39.13)	0 (0.00)	0.15
Intestinal obstruction	4 (8.00)	4 (8.7)	0 (0.00)	0.70

*p*-values were obtained from Fisher’s exact test

*p-values were obtained from the Mann–Whitney test

Bold values indicate statistically significant *p*-values (*p* < 0.05)

Higher mortality was identified with a statistically significant difference in patients with higher body mass index, preoperative vasopressor support, and mechanical ventilation requirement; higher Barthel, SOFA, and APACHE II scores and lower Glasgow scale and fragility index scores.

The most frequent intraoperative findings were the presence of mesenteric ischemia (42%) followed by peritonitis (36%), 2 of the 4 patients without findings during laparotomy survived.

With the statistically significant and clinically relevant variables, a regression model was constructed to evaluate factors associated with mortality at 30 days, finding a statistically significant difference with the APACHE II scale for predicting mortality at 30 days (Table 2). The SOFA, Glasgow, Celiotomy score, vasopressor support requirement, and preoperative mechanical ventilation variables were excluded from the model due to their collinearity with the APACHE II; this can be seen in the Spearman’s Rho correlation coefficient for each of these variables concerning the APACHE II: SOFA: 0.70, Glasgow: 0.74, Celiotomy score 0.55, requirement of preoperative vasopressor support: 0.58 and preoperative mechanical ventilation requirement: 0.66. On the other hand, Barthel Index and Fragility Index variables were excluded from the model because the lower the Barthel Index and the higher the Fragility Index, the lower the mortality rate, which is contrary to what would be expected.

**Table 2:** Negative binomial log

	Coefficient	Robust standard error	*P*-value	IC95%
APACHE II	0.158719	0.007116	**0.02**	0.001–0.029
Body Mass Index	0.118728	0.006754	0.07	0.001–0.025
No intraoperative findings	− 0.6952988	0.442661	0.11	− 1.562–0.172

Bold values indicate statistically significant *p*-values (*p* < 0.05)

We also performed a bivariate analysis which compared patients that died during the first 24 h versus those which survived further than the first 24 h after the surgery considering that this first mortality group had the highest probability for incurring in possible futility and would’ve been the least benefitted from a surgical procedure (Table 3).

**Table 3:** Comparison of demographic, clinical, and surgical characteristics of patients undergoing emergency laparotomy with a mortality risk greater than 75% according to the ACS -NSQIP Surgical Risk Calculator during the first 24 h of outcome

	N (%)	Dead at 24 h	Alive at 24 h	*P*-value
		n = 25 (%)	n = 25 (%)	
Age (median) (IQR) years	82.5 (18.25)	74 (19.50)	83 (15.50)	0.236*
Sex				
Female	17 (34.00)	7 (28.00)	10 (40.00)	0.551
Male	33 (66.00)	18 (72.00)	15 (60.00)	
Body Mass Index (median) (IQR) kg/m^2^	25.00 (9.47)	24.90 (9.4)	26.00 (10.6)	0.479*
Comorbidities				
Diabetes mellitus	11 (22.00)	5 (20.00)	6 (24.00)	1
Arterial hypertension	41 (82.00)	16 (64.00)	25 (100.00)	**0.002**
Chronic obstructive pulmonary disease O_2_ requirement	17 (34.00)	8 (32.00)	9 (36.00)	1
Non-O_2_ requirement obstructive pulmonary disease	4 (8.00)	2 (8.00)	2 (8.00)	1
Chronic kidney disease stage 5	9 (18.00)	4 (16.00)	5 (20.00)	1
Heart failure	15 (30.00)	7 (28.00)	8 (32.00)	1
Acute myocardial infarction < 6 months	2 (4.00)	1 (4.00)	1 (4.00)	0.755
Oncologic disease	12 (24.00)	2 (8.00)	10 (40.00)	**0.018**
Terminal oncologic disease	7 (14.00)	0 (0.00)	7 (28.00)	**0.01**
Stroke with sequelae	5 (10.00)	2 (8.00)	3 (12.00)	1
Charlson Comorbidity Index (median) (IQR) points	6 (2.25)	6 (3.00)	6 (2.50)	0.630*
Barthel Index (median) (IQR) points	65 (31.25)	65 (30.0)	60 (32.5)	0.102
Fragility Index (median) (IQR) points	4.5 (3.00)	4 (3.00)	5 (2.00)	0.232
Vasopressor				
No	15 (30.00)	3 (12.00)	12 (48.00)	**0.012**
Yes	35 (70.00)	22 (88.00)	13 (52.00)	
Mechanical ventilation				
No	23 (46.00)	7 (28.00)	16 (64.00)	**0.022**
Yes	27 (54.00)	18 (72.00)	9 (36.00)	
Glasgow (median) (IQR) points	9 (7.00)	6 (6.00)	12 (7.50)	0.019*
APACHE II (median) (IQR) mortality	36 (15.5)	31.5(12.00)	22 (15.75)	0.078*
SOFA (median) (IQR) points	10 (7.00)	13 (5.00)	8 (6.00)	0.002*
Celiotomy (median) (IQR) points	10 (7.50)	13 (7.75)	8 (5.00)	0.007*
Laboratories (median) (IQR)				
Leukocytes (× 10^3^)	13.45 (10.65)	13.56 (15.08)	13.36 (9.05)	0.522*
Hemoglobin (mg/dl)	11.1 (4.22)	10.6 (3.9)	11.4 (5.45)	0.628*
Platelets (× 10^3^)	170 (184.5)	146 (145)	206 (210)	0.151*
Creatinine (mg/dl)	2.01 (2.23)	2.78 (2.62)	1.76 (1.58)	0.138*
Sodium (mEq/L)	140 (6.5)	140 (8.5)	140 (6.0)	0.593*
Potassium (mEq/L)	4.71 (1.34)	4.8 (1.81)	4.69 (1.61)	0.248*
Glycaemia (mg/dl)	144 (94)	124 (84.5)	189 (98.5)	0.133*
Lactate (mol/L)	5.4 (5.9)	6.55 (6.55)	3.4 (4.12)	0.013*
HCO_3_	16 (8.4)	14.15 (7.55)	17.8 (8.1)	0.022*
pH	7.30 (0.26)	7.20 (0.34)	7.37 (0.19)	0.007*
Pa/FiO_2_	178 (99.5)	162.5 (111.25)	205 (162.5)	0.011*
Intraoperative findings	4 (8.00)	1 (4.00)	3 (12.00)	0.609
No finding	5 (10.00)	5 (20.00)	0 (0.00)	**0.05**
Overall Ischemia	16 (32.00)	11 (44.00)	5 (20.00)	0.128
Ischemia of an isolated segment				
Colitis	3 (6.00)	0 (0.00)	3 (12.00)	0.235
GI Bleeding	9 (18.00)	4 (16.00)	5 (20.00)	1
Peptic ulcer	4 (8.00)	2 (8.00)	2 (8.00)	1
Peritonitis	18 (36.00)	8 (32.00)	10 (40.00)	0.769
Intestinal obstruction	4 (8.00)	1 (4.00)	3 (12.00)	0.609

The *p*-values were obtained from Fisher’s exact test

*The *p*-values were obtained from the Mann–Whitney test

Bold values indicate statistically significant *p*-values (*p* < 0.05)

Patients diagnosed with global mesenteric ischemia had a higher mortality rate during the first 24 h.

A regression model was constructed to evaluate factors associated with mortality at 24 h, finding a statistically significant difference with Pa/FiO2, lactate and global mesenteric ischemia for predicting mortality at 24 h (Table 4).

**Table 4:** Negative binomial log

	Coefficient	Robust standard error	*P*-value	IC95%
Pa/FiO_2_	− 0.0040498	0.0017694	**0.022**	− 0.0075–0.0005
Lactate	0.872884	0.0319346	**0.006**	0.0246–0.1498
Global mesenteric ischemia	0.6555229	0.3131438	**0.036**	0.0417–1.2692

Bold values indicate statistically significant *p*-values (*p* < 0.05)

Postoperative mortality in the series occurred in 46 (92%) of the patients. Twenty-five (54.34%) patients died during the first 24 h, 11 (23.91%) in the next 72 h, and 10 (21.73%) in the next 30 days (Table 5).

**Table 5:** Time at which mortality occurred

Postoperative time	N = 46
< 24 h	25
24–72 h	11
30 days	10

Cause of death in the two patients with no intraoperative findings were due to septic shock secondary to multilobar pneumonia in one patient and cardiogenic shock in the other.

## Discussion

This study evidenced mortality of 92% in the first 30 postoperative days in a series of patients with a risk defined as extreme (≥ 75%) according to the ACS NSQIP Surgical Risk Calculator [8] who underwent emergency laparotomy during a 4-year period. This analysis also showed that more than half of them died during the first 24 h of the postoperative period and that in only two (2/4) of the surviving patients could there be a benefit resulting from surgery, since in the other two survivors of the series there were no findings that retrospectively justified surgery. However, in an individual review of these two surviving cases in which there were positive intraoperative findings during laparotomy, patient demographics were a Barthel Index of 0 points and terminal oncologic disease in one, and a Barthel Index of 55 points and 88 years old in the other. The scant benefit observed in terms of survival invites us to reflect on the possible quantitative and qualitative futility of these procedures.

The median Barthel Index in the series (65 IQR: 31.25) reflects that patients had moderate functional dependence, and a surgical procedure with extreme risk could possibly further deteriorate their functionality.

When comparing the group of patients that died during the first 24 h versus those that survived further than this time period, we could observe that the first group had a higher clinical decline due to a higher rate of vasopressor support, mechanical ventilation, metabolic acidosis and hyperlactatemia; these are all variables that translate into worse scores in the scales used (SOFA, APACHE II, CELIOtomy score and Glasgow). Taking this into account, this information could aide us when deciding if the patient could benefit from the surgical procedure; and because the decision to not perform surgery is always difficult, it should be based on solid data and not only surgeons’ criteria. As a result, even though surgeon’s experience and opinion are valuable it should not be the only tool with which to base a decision: patient’s wishes (if they’re capable of making them known by themselves or through their relatives), clinical and paraclinical variables and morbimortality prediction scores must also be considered in order to not perform futile interventions.

These results provide evidence by confronting actual outcomes in a retrospective series of patients with a risk calculated as extreme by the ACS NSQIP Surgical Risk Calculator and invite us to consider it as a reliable guidance tool in the decision to undertake or avoid performing a potentially futile procedure. Our work also confirms the significant association with mortality to other scores such as APACHE II and SOFA. Although other studies evaluating mortality prediction performance of the CELIOtomy Score or POTTER calculator have also demonstrated their usefulness and other scales such as P-POSSUM or APGAR would also be suitable for an analysis of mortality, they are not useful for preoperative decision making since they require intraoperative variables [4, 10–13].

It is important to identify the possibility of disproportionate or futile surgical procedures and to avoid therapeutic overkill on the part of caregivers and the surgeon. Therapeutic overkill is identified in acts that appear unnecessary, disproportionate, or have no effect other than artificially maintaining life. Therefore, it is legally and ethically acceptable not to perform surgery on a patient if it is considered to overkill or futile. The clinical decision should be directed in the best interest of the patient and respect the patient's previously expressed wishes when they have done so [5, 14]. Preoperative identification of these patients can help guide informed decision-making discussions to avoid unnecessary surgery, minimize pain and suffering, and maximize the quality of time left with loved ones [4, 15].

The disparity between the priorities of patients and those of physicians when proposing their surgery presents communication problems between patients, family, and physicians, partly because in these emergency situations there is no prior relationship of trust between the physician and the patient. In addition, severity of the patient's condition often prevents him/her from participating in decision-making [3]. This context is compounded by the inability of many patients, families, and physicians to recognize the limits of medicine, and drives them to undertake futile treatments [16].

The surgeon is also exposed to being pushed by the patient, family members, or colleagues to perform potentially futile procedures [3]. This attitude is facilitated by the surgeon's ongoing determination in offering healing treatments, by his/her personal inexperience or discomfort in dealing with death, and by ethical and legal concerns [17]. More experienced surgeons better withstand being pressured by external elements, have greater confidence in their futility assessments, and are more comfortable guiding patients and their families away from additional interventions [3, 18, 19]. Various circumstances that put the surgeon at risk of futility are increasingly present due to advances in technology, intensive care, and medicine’s ability to prolong life in increasingly extreme circumstances [5].

Involving support services such as Ethics Committees, palliative care specialists, pastoral care teams and patient representatives in decision-making helps to avoid futility conflicts and improve surgical outcomes [6]. The *Four-Box* method proposes to evaluate four components in decision-making: medical indication, patient preferences, quality of life, and contextual factors [5, 20]. The purpose should not be “to do everything for the patient but to do the best for the patient” [3].

This study recognizes some limitations. The first is that it only included patient registries who were taken for a surgical procedure and does not include the analysis of patients who did not undergo surgery given their extreme risk. It also does not include the analysis of mortality and the comparison of patients with a risk lower than 75% according to the ACS NSQIP Surgical Risk Calculator, in which futile procedures could also be found. And in addition, the retrospective nature and limited sample of the study.

## Conclusions

All available tools should be used to make decisions, with the most reliable and objective information possible to avoid futility and its consequences, especially in patients at extreme risk (mortality risk greater than 75% according to ACS NSQIP Surgical Risk Calculator). The available information should be shared with the patient, their family, or their guardians through an assertive and empathetic communication strategy. It is necessary to insist on a culture of surgical ethics based on reflection and continuous improvement in the care of patients and to know how to accompany them in order to have a proper death.

## Supplementary Information

**Additional file 1.** Raw data.

## Data Availability

You request the data on the following address: ramirezgiraldocamilo@gmail.com.
